# Gastrotomy with Removal of 1149 Foreign Bodies

**Published:** 1913-11

**Authors:** 


Gastrotomy With Removal of 1149 Foreign Bodies. A. C. Matthews of the Napa State Hospital, California, *Calif. State Jour. of Med.,* January, 1913, reports a case in an insane man, aged 31. The foreign bodies included 180 hair pins, 14 closed and 21 broken safety pins, 23 buttons, 13 nails, 425 broken hair pins and pieces of wire, 280 small pins, the total weight being 18 ounces. Vanderwirt and Mills, *Jour. A. M. A.,* January 21, 1911, report a similar case with 1446 foreign bodies removed at necropsy, and refer to a case of Bell of Montreal, in which a hair ball formed a complete cast of the stomach and duodenum.

